# TAVI Success Is More Than Just the Valve: CT‐Derived Sarcopenia as a Major Determinant of Long‐Term Survival

**DOI:** 10.1002/jcsm.70012

**Published:** 2025-07-11

**Authors:** Nikolaos Schörghofer, Christoph Knapitsch, Gretha Hecke, Nikolaus Clodi, Lucas Brandstetter, Crispiana Cozowicz, Matthias Hammerer, Klaus Hergan, Uta C. Hoppe, Bernhard Scharinger, Elke Boxhammer

**Affiliations:** ^1^ Department of Radiology Paracelsus Medical University of Salzburg Salzburg Austria; ^2^ Department of Anesthesiology, Perioperative Medicine and Intensive Care Medicine Paracelsus Medical University Salzburg Austria; ^3^ Department of Internal Medicine II, Division of Cardiology Paracelsus Medical University of Salzburg Salzburg Austria

**Keywords:** computed tomography, psoas muscle area, psoas muscle area index, sarcopenia, TAVI

## Abstract

**Background:**

Sarcopenia, characterized by progressive skeletal muscle loss, is a silent yet powerful marker associated with survival, yet its impact on long‐term outcomes in transcatheter aortic valve implantation (TAVI) remains underestimated. While frailty has been recognized as a main factor of resilience and recovery, the role of muscle integrity is frequently overlooked. This study explores whether computed tomography (CT)‐derived psoas muscle area (PMA) and psoas muscle area index (PMI) are key predictors of post‐TAVI survival.

**Methods:**

A total of 539 patients (mean age 82.0 ± 5.1 years; 49.9% female) undergoing TAVI were analysed in this retrospective, single‐centre study. Sarcopenia was analysed via sex‐specific quartiles of PMA and PMI. Long‐term survival was examined using Kaplan–Meier analysis, univariate and multivariate Cox regression analysis. Interaction terms were introduced to assess whether the association between sarcopenia and survival differed by age, sex, renal function and anaemia status.

**Results:**

Sarcopenia emerged as a predictor of long‐term mortality (HR = 1.52, *p* = 0.011 for PMA; HR = 1.55, *p* = 0.008 for PMI) after TAVI. Notably, younger patients (< 80 years) with sarcopenia faced double the mortality risk (HR = 2.48, *p* = 0.001 for PMA; HR = 2.55, *p* = 0.001 for PMI), whereas in older patients, the association was weaker. In women, who typically show a post‐TAVI survival advantage, sarcopenia reduced this benefit (HR = 1.75, *p* = 0.020 for PMA; HR = 1.88, *p* = 0.008 for PMI). The most striking finding was the synergistic effect of sarcopenia and chronic kidney disease (CKD), resulting in a threefold increase in mortality risk (HR = 2.82, *p* = 0.027 for PMA; HR 3.14, *p* = 0.011). After multivariate adjustment, sarcopenia remained a strong, independent predictor of long‐term mortality (HR = 1.58, *p* = 0.009 for PMA; HR = 1.49, *p* = 0.024 for PMI), reinforcing its clinical relevance in TAVI risk stratification.

**Conclusion:**

Our study suggests that sarcopenia is not just a passive bystander, but may serve as a marker associated with long‐term mortality in TAVI patients, especially in younger individuals, women and those with CKD or anaemia. Since muscle mass predicts survival and is potentially modifiable, assessing and intervening against sarcopenia before and after TAVI could represent a clinical priority in patients with aortic valve stenosis. This study underscores the importance of a more nuanced approach—not merely focusing on valve replacement, but on strengthening patient‐centred care.

## 1 Introduction

Sarcopenia, the progressive and generalized loss of skeletal muscle mass and function, is more than a hallmark of aging [1]—it is a critical, yet underappreciated, marker of reduced resilience in patients undergoing major medical interventions [2]. With its roots in chronic inflammation, metabolic dysregulation and neuromuscular decline, sarcopenia does not merely reflect biological aging but serves as a dynamic indicator of physiological vulnerability [3, 4]. Nowhere has this become more evident based on trial data than in patients facing transcatheter aortic valve implantation (TAVI), where preexisting muscle deterioration may influence the body's ability to withstand and recover from procedural stress [5].

Despite the growing recognition of sarcopenia as a key factor influencing health outcomes, its role in long‐term recovery and survival following TAVI remains largely unexplored. Traditional risk assessment tools often overlook the profound impact of muscle depletion on systemic health, focusing instead on cardiovascular parameters [6]. However, skeletal muscle is not just about movement; it is a metabolic hub, a reservoir of amino acids for immune responses and a central player in inflammation regulation. When this critical tissue deteriorates, patients may enter a downward spiral of worsening frailty, prolonged functional impairment and heightened susceptibility to complications [7].

Computed tomography (CT)‐derived measurements of muscle mass, particularly the psoas muscle area (PMA) and the psoas muscle area index (PMI), offer an objective and reproducible window into the patient's muscular integrity [8, 9]. These imaging biomarkers have been linked to adverse outcomes in surgical and critically ill populations [10, 11, 12]. While short‐term associations between low muscle mass and procedural risk have been often described in TAVI [5, 13, 14, 15], the long‐term trajectory of sarcopenia and its role in shaping postprocedural recovery in different subgroups has never been systematically analysed.

In this study, we take a bold step toward redefining how sarcopenia is perceived in the TAVI landscape. By analysing 5‐year follow‐up data, we move beyond static periprocedural assessments to explore whether baseline muscle mass is associated not only with survival but also the capacity for post‐TAVI rehabilitation.

We hypothesize that CT‐derived PMA and PMI are not just passive reflections of frailty but may serve as indicators of post‐TAVI resilience. Our study not only quantifies the burden of sarcopenia but also identifies patient subgroups who are particularly vulnerable—offering a potential foundation for targeted exercise, nutrition and/or medication‐based interventions. This study connects muscle health with cardiovascular interventions, drawing attention to skeletal muscle as a meaningful contributor to post‐TAVI outcomes‐alongside the established focus on valve function.

## 2 Material and Methods

### 2.1 Study Design and Population

This was a retrospective, single‐centre observational cohort study conducted at Paracelsus Medical University Hospital Salzburg. It examined data from patients who underwent TAVI between January 2016 and June 2022. The initial cohort comprised 585 patients with severe aortic stenosis (AS). Forty‐six patients were excluded due to missing CT data. As a result, the final analysis included 539 patients, of whom 269 were women and 270 were men.

### 2.2 Data Extraction

Patient data were extracted from the ORBIS electronic medical records system (Agfa Healthcare, Version 08043301.04110DACHL) and the Uniklinikum Salzburg medical archive (Krankengeschichtsarchiv System, Softworx by Andreas Schwab TM, 2008). The extracted data included patients' medical records, admission and discharge documents and reports from echocardiographic and imaging examinations performed during hospitalization for the TAVI procedure.

### 2.3 CT Angiography Image Acquisition, Image Analysis and Measurement of PMA

All patients underwent routine preprocedural, ECG‐gated CT scanning, covering the area from the lung apex to the proximal femoral arteries to obtain essential measurements for TAVI planning. Scans were performed using either a 256‐slice or a 128‐slice dual‐source CT scanner (Revolution, General Electric Healthcare, IL, USA, or Somatom Definition AS+, Siemens Healthcare, Erlangen, Germany). Tube voltage (80–120 kVp) was adjusted based on patient size, with active tube current modulation applied. Contrast enhancement was achieved via a bolus‐tracking technique, administering 100 mL of a nonionic iodinated contrast agent, followed by 70 mL of saline at a flow rate of 3.5–5 mL/s.

**FIGURE 1:**
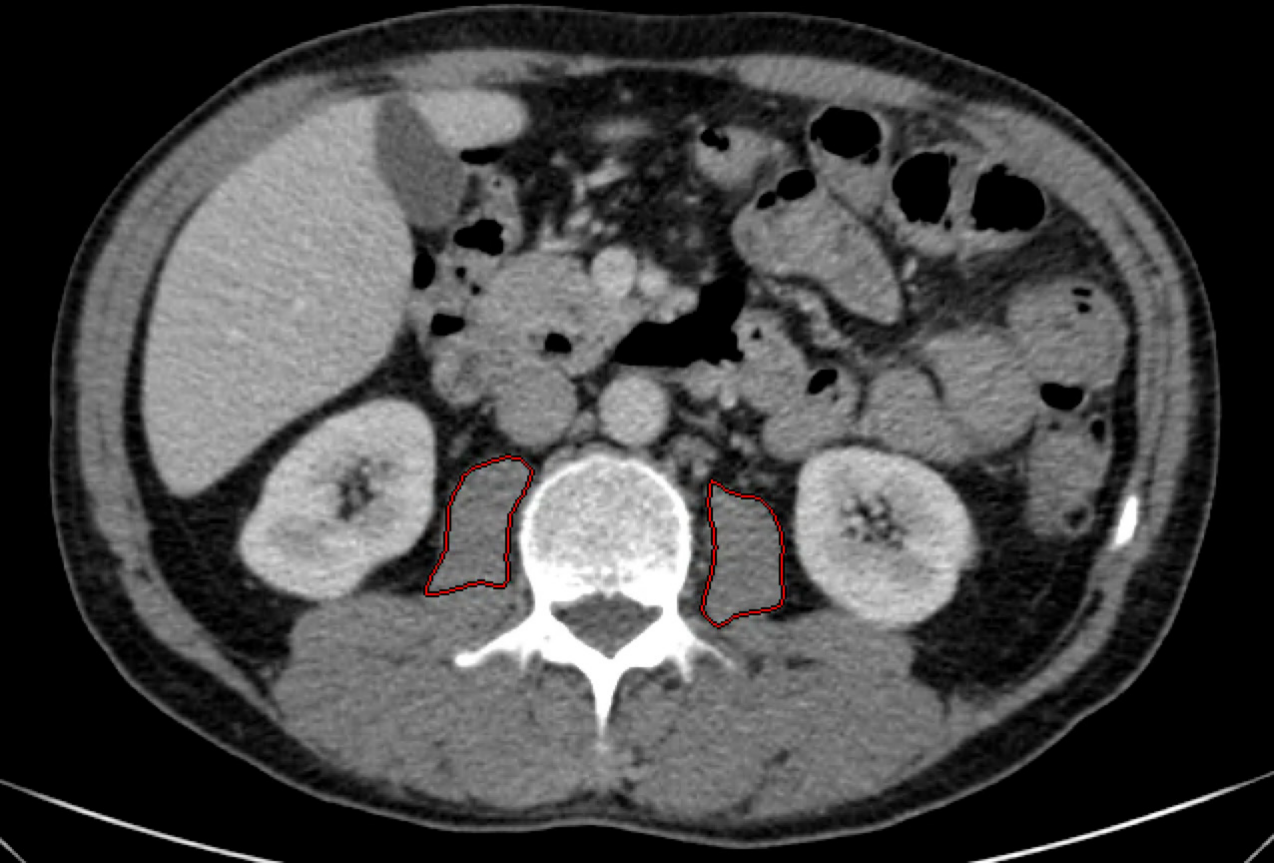
Radiological measurement of PMA. Figure 1 demonstrates the assessment of bilateral psoas muscle cross‐sectional areas through manual delineation of their contours on an axial CT scan. The combined area of both muscles, normalized to BSA, was utilized to derive the PMI. Abbreviations: BSA, body surface area; CT, computed tomography; PMA, psoas muscle area; PMI, psoas muscle area index; Q, quartile.

CT imaging data were analysed using a soft tissue kernel with a slice thickness of 3 mm and a reconstruction interval of 2 mm. The PMA (mm^2^) was measured at the level of the superior endplate of the third lumbar vertebra (L3) by manually outlining both the right and left psoas muscles (Figure 1). The total PMA was calculated as the sum of both sides, and normalization to body surface area (BSA) yielded the indexed PMA (PMI, mm^2^/m^2^). All measurements were conducted using a picture archiving and communication system (PACS, Workstation, Impax; Agfa, Mortsel, Belgium). BSA was determined using the DuBois formula: BSA = 0.007184 × height^0.725^ × weight^0.425^.

### 2.4 TAVI Procedure

All patients underwent transfemoral TAVI with second‐ or third‐generation valve systems, namely, the CoreValve Evolut R and CoreValve Evolut Pro (Medtronic Inc., Minneapolis, MN, USA) due to severe AS, following standard procedural guidelines. Preprocedural imaging, including transthoracic echocardiography (TTE), CTA and—when necessary—transesophageal echocardiography (TEE), was utilized to confirm the diagnosis and aid in procedure planning, particularly for selecting the appropriate valve size.

### 2.5 Outcomes

The primary outcome of this study was long‐term overall survival, tracked from the date of the TAVI procedure with a maximum follow‐up period of 60 months. Mortality data were systematically collected from medical records, follow‐up phone calls to primary care physicians, direct contact with family members and death certificates.

### 2.6 Statistical Analysis

Statistical analyses were performed using SPSS (Version 25.0, SPSS Inc., USA) and R (version 4.2.3, R Foundation for Statistical Computing, Vienna, Austria).

To evaluate the correlation between PMA and body composition parameters, Pearson's correlation coefficient (*r*) was calculated. In our study, PMA (mm^2^) showed a stronger correlation with BSA (m^2^) based on the DuBois formula (*r* = 0.593; *p* < 0.001) than with body mass index (BMI, kg/m^2^) (*r* = 0.357; *p* < 0.001). Consequently, PMA was normalized to BSA, resulting in the indexed PMA (PMI, mm^2^/m^2^). Given the gender dependency of PMI, it was divided into sex‐specific quartiles, with patients in the lowest quartile classified as sarcopenic.

A priori power analysis was conducted using G*Power (Version 3.1) to determine the required sample size for detecting a moderate effect (Cohen's d = 0.5) with a two‐tailed independent *t*‐test. Given an alpha level of 0.05 and a desired power of 0.95, the analysis indicated that a total sample size of 280 patients (70 with sarcopenia and 210 without) would be necessary. However, with the actual study cohort of 136 vs. 403 patients, the achieved power is > 0.99, ensuring a highly robust statistical analysis with a low risk of Type II error.

Normal distribution of variables was assessed using the Kolmogorov–Smirnov test. Normally distributed continuous variables were reported as mean ± standard deviation (SD) and compared using an unpaired Student's *t*‐test. Nonnormally distributed continuous variables were presented as median and interquartile range (IQR) and analysed using the Mann–Whitney *U* test. Categorical variables were expressed as frequencies and percentages, with group comparisons performed using the chi‐square test.

To evaluate differences in long‐term survival between patients with and without radiologically confirmed sarcopenia, Kaplan–Meier survival curves were generated, and differences were assessed using the log‐rank test.

To assess the association between sarcopenia and long‐term mortality, we performed univariate and multivariate Cox proportional hazards regression analyses. Hazard ratios (HR) with 95% confidence intervals (CI) were calculated to quantify the effect of sarcopenia on survival outcomes. For the univariate analysis, we first evaluated the impact of sarcopenia (assessed by both PMA and PMI) on mortality in the overall cohort. Additionally, subgroup analyses were conducted by stratifying patients based on clinically relevant characteristics, including age, sex, left ventricular ejection fraction, stroke volume index (SVi), New York Heart Association (NYHA) functional class, renal function, body mass index (BMI) and anaemia status (Hb < 12 g/dL for females and < 13 g/dL for males). For the multivariate Cox regression, a stepwise backward elimination approach was applied, where all relevant variables were initially included in the model. Three hierarchical models were constructed to account for potential confounders. Model 1 included basic adjustments for age and sex. Model 2 was additionally adjusted for cardiac factors such as left ventricular function (LVEF) and SVi. Model 3 further incorporated additional clinical variables, including creatinine, BMI and haemoglobin, representing the “metabolic” adjusted model.

To further explore effect modifications, interaction terms were introduced into the multivariate Cox models to test whether the impact of sarcopenia on mortality differed between subgroups (e.g., sarcopenia × age, sarcopenia × sex and sarcopenia × LVEF). The proportional hazards assumption was verified using Schoenfeld residuals, and all models were tested for multicollinearity.

A two‐tailed *p*‐value < 0.050 was considered statistically significant.

## 3 Results

### 3.1 Baseline Characteristics and Cut‐Off Values of Sarcopenia in TAVI

**FIGURE 2:**
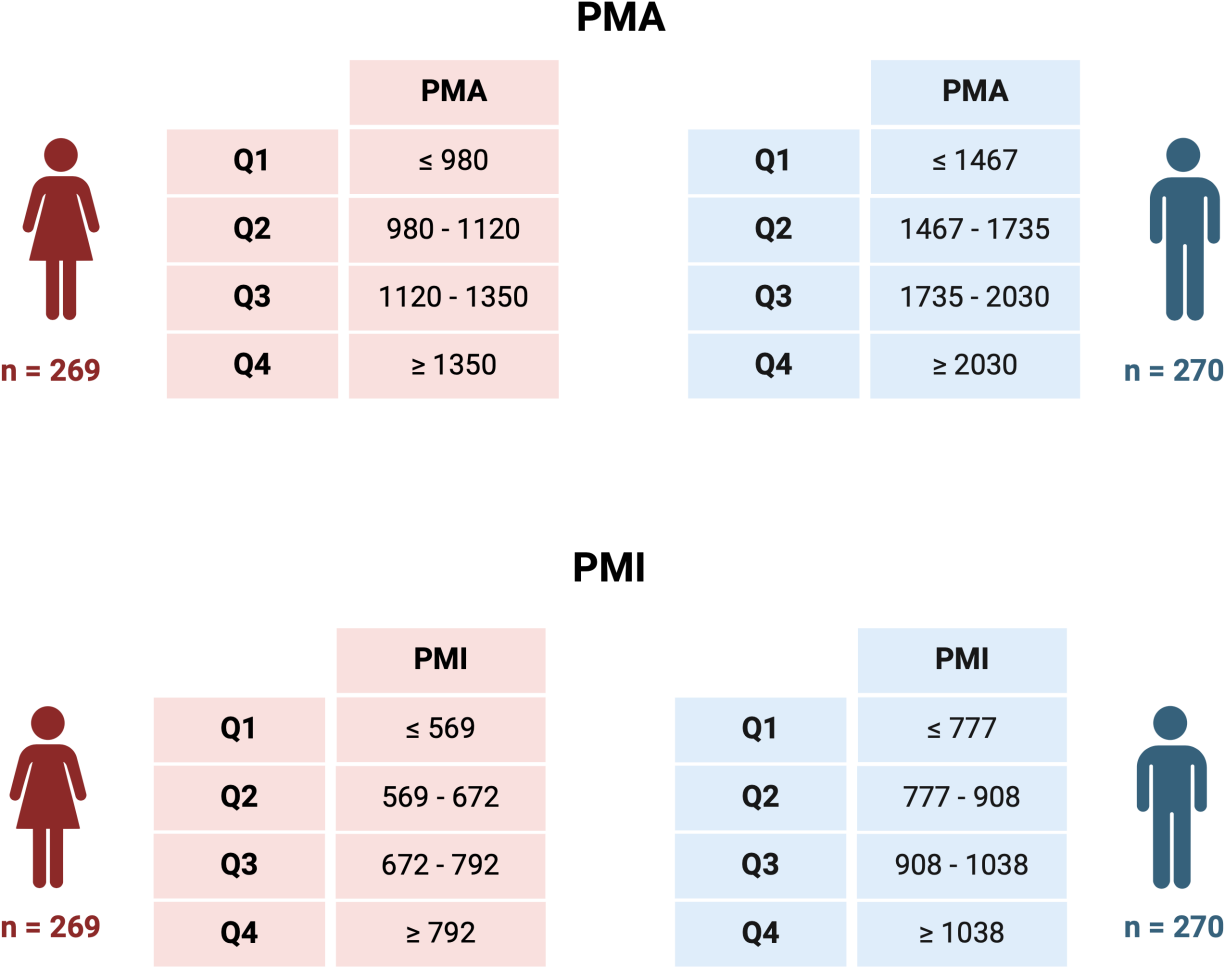
Sex‐specific quartile distribution of PMA and PMI, used for sarcopenia classification in the study cohort. Abbreviations: PMA, psoas muscle area; PMI, psoas muscle area index; Q, quartile.

The study cohort consisted of 539 patients (Table 1) with a mean age of 82.0 ± 5.1 years and an equal distribution of sexes (269 female and 270 male). As shown in Figure 2 sarcopenia classification was determined using the first quartile (Q1) of the PMA and PMI values for each sex. The sarcopenia cut‐off values were PMA ≤ 980 mm^2^ and PMI ≤ 569 mm^2^/m^2^ for females and PMA ≤ 1467 mm^2^ and PMI ≤ 777 mm^2^/m^2^ for males. Participants with values within these respective ranges were classified as sarcopenic. By these definitions, sarcopenia was present in 25.2% of patients.

**TABLE 1:** Baseline characteristics of study cohort.

	Total	Sarcopenia PMA	No sarcopenia PMA	*p* PMA	sarcopenia PMI	No sarcopenia PMI	*p* PMI
**No. (%)**							
Total	539 (100.0)	136 (25.2)	403 (74.8)	—	136 (25.2)	403 (74.5)	—
Sex							
Female	269 (49.9)	68 (50.0)	201 (49.9)	0.980	68 (50.0)	201 (49.9)	0.980
Male	270 (50.1)	68 (50.0)	202 (50.1)		68 (50.0)	202 (50.1)	
Age							
≤ 80	209 (38.8)	41 (30.1)	168 (41.7)	0.017	41 (30.1)	168 (41.7)	0.017
> 80	330 (61.2)	95 (69.9)	235 (58.3)		95 (69.9)	235 (58.3)	
BMI							
< 25.0 kg/m^2^	258 (47.9)	82 (60.3)	176 (43.7)	< 0.001	69 (50.7)	189 (46.9)	0.495
≥ 25.0 kg/m^2^	281 (52.1)	54 (39.7)	227 (56.3)		67 (49.3)	214 (53.1)	
NYHA							
< III	317 (58.8)	80 (58.8)	237 (58.8)	0.946	77 (56.6)	240 (59.6)	0.580
≥ III	222 (41.2)	56 (41.2)	166 (41.2)		59 (43.4)	163 (40.4)	
Diabetes mellitus	141 (26.2)	32 (23.5)	109 (27.9)	0.420	37 (27.2)	104 (25.8)	0.748
Arterial hypertension	467 (86.6)	116 (85.3)	351 (87.1)	0.548	113 (83.1)	354 (87.8)	0.219
CHD	274 (50.8)	67 (48.5)	207 (51.3)	0.755	63 (46.3)	211 (52.3)	0.469
AF	192 (35.6)	53 (39.0)	139 (34.5)	0.346	55 (40.4)	137 (34.0)	0.175
PAOD	48 (8.9)	7 (5.1)	41 (10.2)	0.075	6 (4.4)	42 (10.4)	0.033
COPD	58 (10.8)	19 (14.0)	39 (9.7)	0.162	18 (13.2)	40 (9.9)	0.281
MI—prehistory	39 (7.2)	9 (6.6)	30 (7.4)	0.748	8 (5.9)	31 (7.7)	0.481
Cardiac surgery—prehistory	19 (3.5)	4 (2.9)	15 (3.7)	0.669	4 (2.9)	15 (3.7)	0.669
Malignancy—prehistory	97 (18.0)	23 (16.9)	74 (18.4)	0.703	23 (16.9)	74 (18.4)	0.703
Stroke—prehistory	44 (8.2)	7 (5.1)	37 (9.2)	0.137	7 (5.1)	37 (9.2)	0.137
LVEF							
≤ 40%	64 (11.9)	16 (11.8)	48 (11.9)	0.965	17 (12.5)	47 (11.7)	0.579
41%–54%	119 (22.1)	39 (28.7)	80 (19.9)	0.037	35 (25.7)	84 (20.8)	0.033
≥ 55%	356 (66.0)	81 (59.5)	275 (68.2)	0.072	84 (61.8)	272 (67.5)	0.085
SVi							
< 35 mL/m^2^	125 (23.2)	30 (22.1)	95 (23.6)	0.770	35 (25.6)	90 (22.3)	0.377
≥ 35 mL/m^2^	414 (76.8)	106 (77.9)	308 (76.4)		101 (74.4)	313 (77.7)	
Creatinine							
< 1.5 mg/dL	469 (87.0)	125 (91.9)	344 (85.4)	0.020	124 (91.2)	345 (85.6)	0.087
≥ 1.5 mg/dL	70 (13.0)	11 (8.1)	59 (14.6)		12 (8.8)	58 (14.4)	
Anaemia							
HB < 12/13 g/dL in female/male	213 (39.5)	57 (41.9)	156 (38.7)	0.509	55 (40.4)	158 (39.2)	0.799
HB ≥ 12/13 g/dL in female/male	326 (60.4)	79 (58.1)	247 (61.3)		81 (59.6)	245 (60.8)	
**Mean ± SD**							
Age (years)	82.0 ± 5.1	82.8 ± 5.0	81.7 ± 5.2	0.024	82.7 ± 5.0	81.8 ± 5.2	0.075
Height (cm)	167.8 ± 8.8	167.4 ± 8.3	167.9 ± 9.0	0.560	168.6 ± 7.9	167.5 ± 9.1	0.208
Weight (kg)	72.6 ± 14.5	68.2 ± 10.8	74.2 ± 15.3	< 0.001	71.5 ± 11.2	73.0 ± 15.5	0.208
BMI (kg/m^2^)	25.7 ± 4.4	24.3 ± 3.2	26.2 ± 4.6	< 0.001	25.1 ± 3.4	25.9 ± 4.6	0.035
BSA (m^2^)	1.8 ± 0.2	1.7 ± 0.2	1.8 ± 0.2	0.001	1.8 ± 0.2	1.8 ± 0.2	0.997
LVEF (%)	53.0 ± 9.9	52.4 ± 10.6	53.2 ± 9.6	0.451	52.7 ± 11.0	53.1 ± 9.5	0.655
LVEDD (mm)	46.4 ± 6.7	46.0 ± 7.5	46.5 ± 6.5	0.472	45.9 ± 7.5	46.6 ± 6.5	0.370
IVSd (mm)	13.4 ± 2.2	13.4 ± 2.3	13.5 ± 2.2	0.715	13.5 ± 2.4	13.4 ± 2.1	0.761
AV Vmax (m/s)	4.4 ± 0.6	4.4 ± 0.5	4.4 ± 0.6	0.710	4.4 ± 0.6	4.4 ± 0.5	0.448
AV MAX (mmHg)	77.3 ± 19.1	77.7 ± 19.1	77.1 ± 19.1	0.751	78.4 ± 19.9	76.9 ± 18.8	0.417
AV MPG (mmHg)	45.6 ± 12.1	45.8 ± 12.2	45.5 ± 12.0	0.801	46.1 ± 12.6	45.4 ± 11.9	0.566
LVOT (mm)	21.0 ± 2.4	20.9 ± 2.4	21.1 ± 2.4	0.385	21.1 ± 2.5	21.0 ± 2.4	0.558
SV (mL)	84.4 ± 27.9	86.0 ± 28.9	83.9 ± 27.6	0.449	86.2 ± 29.5	83.8 ± 27.4	0.394
SVi (mL/m^2^)	46.8 ± 15.8	49.2 ± 16.9	46.0 ± 15.4	0.043	48.2 ± 17.1	46.3 ± 15.4	0.241
**Median ± IQR**							
Creatinine (mg/dL)	1.0 ± 0.5	1.0 ± 0.4	1.0 ± 0.5	0.081	1.0 ± 0.4	1.0 ± 0.5	0.359
HK (%)	38.0 ± 6.5	38.1 ± 6.5	38.0 ± 6.4	0.815	38.3 ± 6.5	37.9 ± 6.3	0.792
HB (g/dL)	12.9 ± 2.1	12.8 ± 2.3	12.9 ± 2.1	0.799	12.9 ± 2.2	12.8 ± 2.1	0.791
CK (U/L)	83.0 ± 68.0	73.5 ± 51.5	85.0 ± 69.0	0.002	72.8 ± 57.6	85.0 ± 68.0	0.013
PMA (mm^2^)	1430.0 ± 710.0	970.0 ± 480.0	1620.0 ± 680.0	< 0.001	1000.0 ± 492.5	1600.0 ± 690.0	< 0.001
PMI (mm^2^/m^2^)	787.4 ± 298.7	575.9 ± 214.4	857.9 ± 265.8	< 0.001	559.9 ± 217.1	854.9 ± 264.8	< 0.001

Abbreviations: AF, atrial fibrillation; AV MAX, aortic valve maximum pressure gradient; AV MPG, aortic valve mean pressure gradient; AV Vmax, aortic valve maximum velocity; BSA, body surface area; BMI, body mass index; CHD, coronary heart disease; CK, creatine kinase; COPD, chronic obstructive pulmonary disease; HB, haemoglobin; HK, haematocrit; IVSd, interventricular septal diameter in diastole; LVEF, left ventricular ejection fraction; LVEDD, left ventricular end‐diastolic diameter; LVOT, left ventricular outflow tract; MI, myocardial infarction; NYHA, New York Heart Association Functional Classification; PAOD, peripheral arterial occlusive disease; PMA, psoas muscle area; PMI, psoas muscle area index; SV, stroke volume; SVi, stroke volume index.

While PMA alone showed significant differences between sarcopenic and nonsarcopenic individuals concerning clinical body composition parameters, indexing muscle mass to BSA through PMI appeared to attenuate these disparities, providing a more standardized assessment. In the PMA‐defined groups, sarcopenic patients tended to be slightly older than nonsarcopenic individuals (82.8 ± 5.0 vs. 81.7 ± 5.2 years, *p* = 0.024), suggesting a potential age‐related decline in absolute muscle mass. However, when indexed to BSA using PMI, this age difference was no longer statistically significant (82.7 ± 5.0 vs. 81.8 ± 5.2 years, *p* = 0.075). Beyond muscle mass, the clinical characteristics were largely comparable between groups, with no major differences in key cardiovascular comorbidities such as arterial hypertension or coronary heart disease.

### 3.2 PMA/PMI and Long‐Term Mortality

**FIGURE 3:**
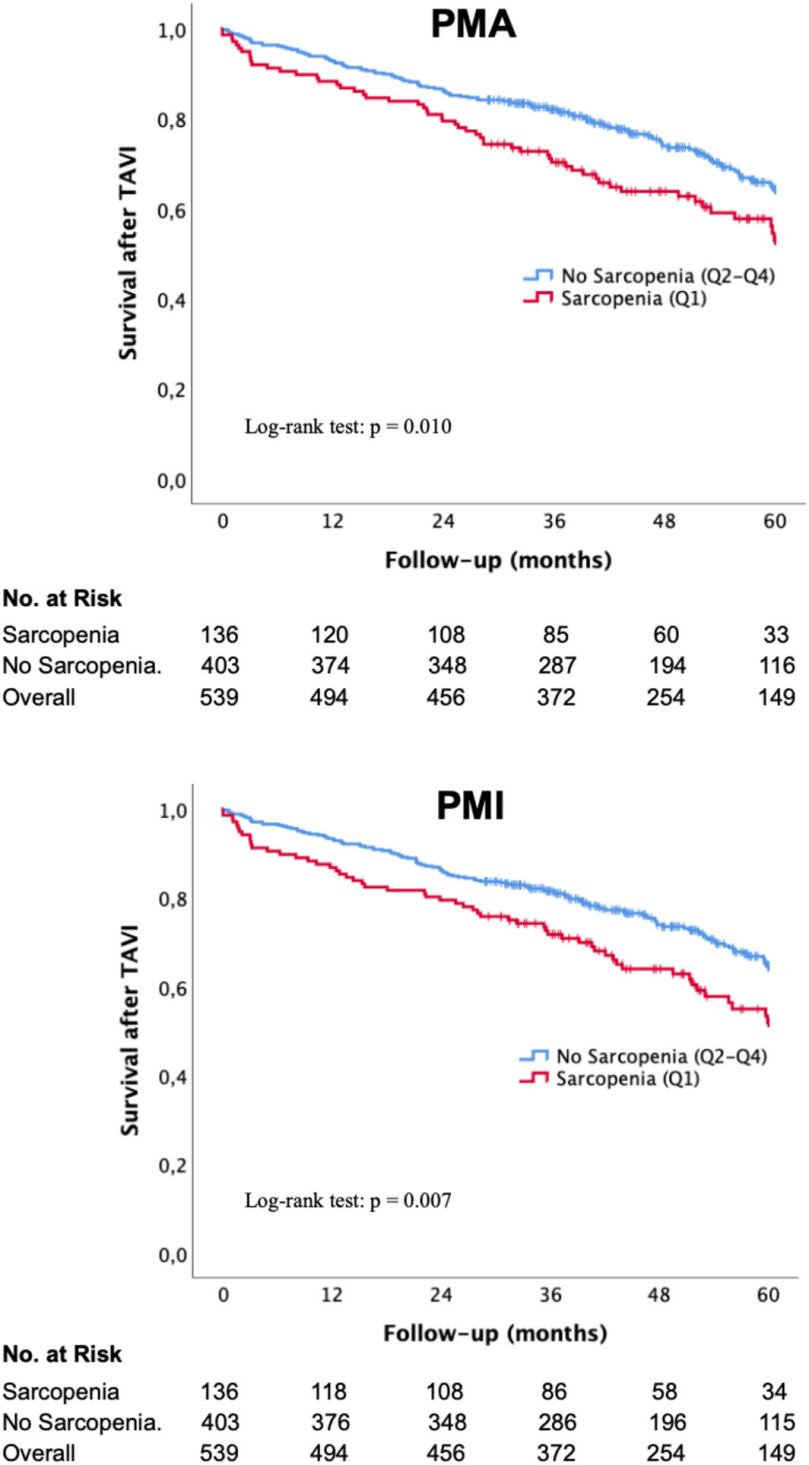
Kaplan–Meier survival curves for PMA and PMI with numbers at risk and corresponding log‐rank tests, comparing long‐term survival in sarcopenic and nonsarcopenic patients. Abbreviations: no., number; PMA, psoas muscle area; PMI, psoas muscle area index; Q, quartile; TAVI, transcatheter aortic valve implantation.

Patients were followed for a median duration of 46.1 ± 27.3 months. Figure 3 illustrates the association between PMA, PMI and long‐term mortality following TAVI. The Kaplan–Meier survival curves demonstrate significantly lower survival rates in sarcopenic patients compared to nonsarcopenic individuals. In both PMA‐ and PMI‐based analyses, the sarcopenic group consistently showed lower survival rates at all time points. The number of at‐risk patients steadily declined, with a more pronounced survival drop observed in the sarcopenic cohort over time. The log‐rank tests confirm statistically significant differences between sarcopenic and nonsarcopenic patients (PMA: *p* = 0.010; PMI: *p* = 0.007), supporting an association between reduced muscle mass and increased long‐term mortality.

### 3.3 Univariate Cox Regression of PMA/PMI and Subgroup Analyses

**FIGURE 4:**
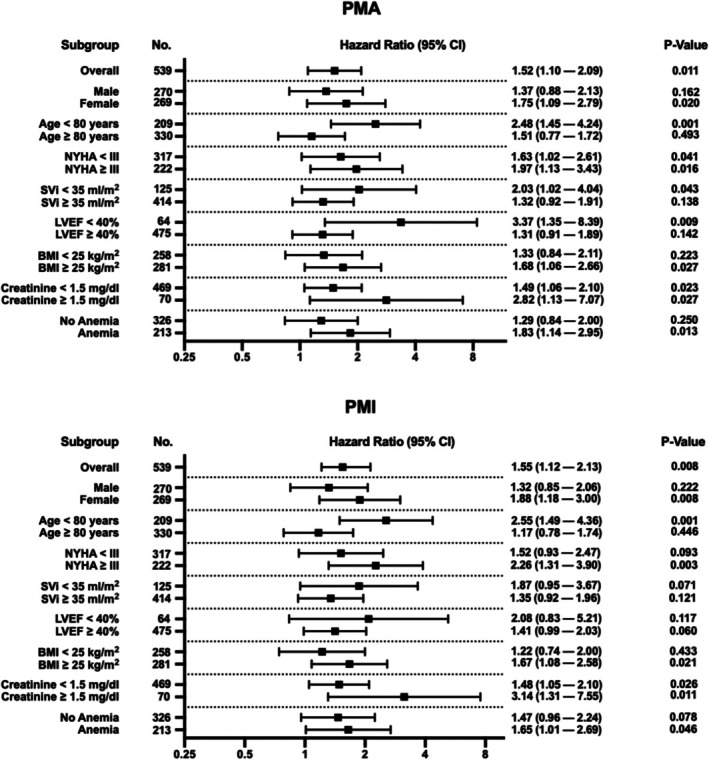
Univariate Cox regression analysis of sarcopenia (PMA and PMI), showing hazard ratios and 95% confidence intervals for different patient subgroups. Abbreviations: BMI, body mass index; CI, confidence interval; LVEF, left ventricular ejection fraction; no., number; NYHA, New York Heart Association Functional Classification; PMA, psoas muscle area; PMI, psoas muscle area index; SVi, stroke volume index.

Figure 4 presents the univariate Cox regression analysis, demonstrating the significant association between sarcopenia and long‐term mortality in both the overall cohort and specific patient subgroups. In the total study population, sarcopenia, as assessed by PMA and PMI, was significantly linked to increased mortality risk (HR = 1.52, 95% CI: 1.10–2.09, *p* = 0.011 for PMA; HR = 1.55, 95% CI: 1.12–2.13, *p* = 0.008 for PMI).

Importantly, the impact of sarcopenia varied across different patient subgroups. Age‐stratified analysis revealed that younger patients (< 80 years) with sarcopenia had a significantly higher risk of mortality (HR = 2.48, 95% CI: 1.45–4.24, *p* = 0.001 for PMA; HR = 2.55, 95% CI: 1.49–4.36, *p* = 0.001 for PMI) In contrast, in older patients (≥ 80 years), the association was weaker and not statistically significant. Sex‐specific analysis further underscored the varying impact of sarcopenia, with a particularly strong association observed in female patients (HR = 1.75, 95% CI: 1.09–2.79, *p* = 0.020 for PMA; HR = 1.88, 95% CI: 1.18–3.00, *p* = 0.008 for PMI). In male patients, the relationship between sarcopenia and mortality was weaker, suggesting potential sex‐based differences in muscle mass reserves, functional resilience or other underlying physiological factors influencing survival after TAVI.

Beyond age and sex, additional subgroup analyses examined factors such as cardiac function (LVEF < 40%, SVi < 35 mL/m^2^), heart failure severity (NYHA stage ≥ III), body composition (BMI < 25 kg/m^2^), renal function (creatinine ≥ 1.5 mg/dL) and anaemia status (defined as haemoglobin < 13 g/dL in male and < 12 g/dL in female patients). Among these in PMA‐defined sarcopenia, patients with LVEF < 40%, SVi < 35 mL/m^2^ and anaemia showed a particularly strong association between sarcopenia and mortality, indicating that reduced muscle mass may have an additive negative effect in those with preexisting functional impairments.

### 3.4 Multivariate Cox Regression

The results of the multivariate Cox regression analysis, presented in Table 2, demonstrate a consistent association between sarcopenia and long‐term mortality, even after adjusting for various confounding factors. In the basic model (Model 1), which included age and sex as covariates, sarcopenia defined by PMA was associated with a 52% increased risk of mortality (HR = 1.52, 95% CI: 1.11–2.10, *p* = 0.010), while PMI‐Sarcopenia showed a similar effect with a 55% higher mortality risk (HR = 1.55, 95% CI: 1.13–2.14, *p* = 0.007). Although age did not reach statistical significance (HR = 1.05, *p* = 0.540), female patients exhibited a reduced risk compared to male patients (HR = 0.73, *p* = 0.036 for PMA; *p* = 0.037 for PMI).

**TABLE 2:** Multivariate Cox regression analysis of sarcopenia (PMA and PMI).

PMA						
	Model 1 HR (95% CI)	*p*	Model 2 HR (95% CI)	*p*	Model 3 HR (95% CI)	*p*
**PMA‐Sarcopenia**	1.52 (1.11–2.10)	0.010	1.44 (1.03–2.03)	0.033	1.58 (1.12–2.22)	0.009
**Age**	1.05 (0.90–1.22)	0.540	1.11 (0.94–1.30)	0.220	1.03 (0.88–1.22)	0.700
**Sex (female)**	0.73 (0.54–0.98)	0.036	0.75 (0.55–1.04)	0.082	0.94 (0.67–1.31)	0.698
**LVEF**	—	—	0.98 (0.83–1.16)	0.842	1.02 (0.87–1.22)	0.787
**SVi**	—	—	0.95 (0.81–1.11)	0.507	0.98 (0.84–1.15)	0.795
**BMI**	—	—	—	—	0.98 (0.82–1.17)	0.831
**Creatinine**	—	—	—	—	1.51 (1.28–1.77)	< 0.001
**Haemoglobin**	—	—	—	—	1.00 (0.85–1.17)	0.948

PMI						
	Model 1 HR (95% CI)	*p*	Model 2 HR (95% CI)	*p*	Model 3 HR (95% CI)	*p*
**PMI‐Sarcopenia**	1.55 (1.13–2.14)	0.007	1.34 (0.98–1.95)	0.063	1.49 (1.05–2.10)	0.024
**Age**	1.05 (0.90–1.23)	0.517	1.11 (0.95–1.31)	0.197	1.03 (0.87–1.23)	0.744
**Sex (female)**	0.73 (0.54–0.98)	0.037	0.76 (0.55–1.04)	0.086	0.94 (0.67–1.32)	0.725
**LVEF**	—	—	0.98 (0.83–1.16)	0.813	1.02 (0.87–1.21)	0.798
**SVi**	—	—	0.96 (0.82–1.11)	0.555	0.98 (0.84–1.15)	0.840
**BMI**	—	—	—	—	0.95 (0.80–1.12)	0.545
**Creatinine**	—	—	—	—	1.50 (1.27–1.76)	< 0.001
**Haemoglobin**	—	—	—	—	0.99 (0.84–1.17)	0.906

*Note:* Model 1—basic model adjusted for age and sex. Model 2—cardiac model additionally adjusted for LVEF and SVi. Model 3—metabolic model additionally adjusted for haemoglobin, BMI and creatinine.

Abbreviations: BMI, body mass index; CI, confidence interval; creatinine, serum creatinine; haemoglobin, haemoglobin concentration; HR, hazard ratio; LVEF, left ventricular ejection fraction; PMA, psoas muscle area; PMI, psoas muscle area index; SVi, stroke volume index.

After further adjusting for cardiac parameters (Model 2), including LVEF and SVi, the association between PMA‐Sarcopenia and mortality remained significant, albeit slightly attenuated (HR = 1.44, *p* = 0.033). In contrast, the effect of PMI‐Sarcopenia weakened and lost statistical significance (HR = 1.34, *p* = 0.063). Neither LVEF (HR = 0.98, *p* = 0.842) nor SVi (HR = 0.95, *p* = 0.507) showed a relevant impact on survival in this model, suggesting that the relationship between sarcopenia and mortality was not merely a reflection of underlying cardiac dysfunction.

In a further adjusted model (Model 3), which incorporated renal function, BMI and haemoglobin levels, sarcopenia remained an independent predictor of mortality, with PMA‐Sarcopenia showing an even stronger association (HR = 1.58, 95% CI: 1.12–2.22, *p* = 0.009) and PMI‐Sarcopenia regaining statistical significance (HR = 1.49, 95% CI: 1.06–2.11, *p* = 0.024). Among the additional variables, elevated creatinine levels emerged as a key predictor of mortality, with a HR of 1.51 (*p* < 0.001 for PMA; *p* < 0.001 for PMI), emphasizing the role of renal impairment in overall prognosis. In contrast, BMI (HR = 0.98, *p* = 0.831) and haemoglobin levels (HR = 1.00, *p* = 0.948) did not show significant associations, suggesting that the adverse effects of sarcopenia are independent of body composition or anaemia status.

### 3.5 Interaction Terms

**FIGURE 5:**
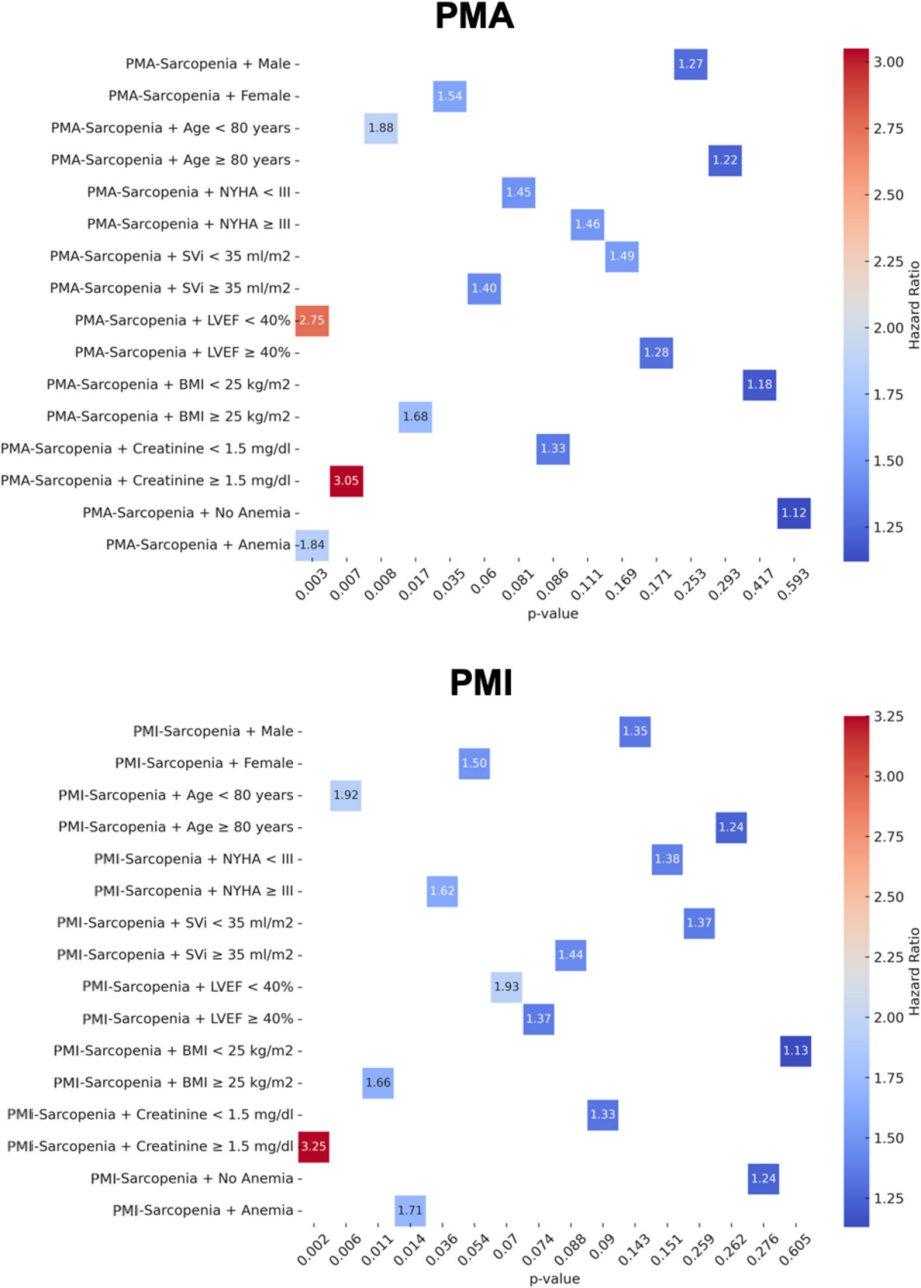
Heat maps illustrating interaction terms between sarcopenia (PMA and PMI), renal function, anaemia, age and sex, highlighting their combined impact on long‐term survival. Abbreviations: BMI, body mass index; LVEF, left ventricular ejection fraction; NYHA, New York Heart Association Functional Classification; PMA, psoas muscle area; PMI, psoas muscle area index; SVi, stroke volume index.

The heat map analysis (Figure 5) illustrates the impact of sarcopenia (assessed by PMA and PMI) on long‐term mortality across various patient subgroups.

#### 3.5.1 Sex‐Specific Differences

The association between sarcopenia and mortality was more pronounced in female patients, with PMA‐Sarcopenia showing an HR of 1.54 (95% CI: 1.03–2.31, *p* = 0.035) and PMI‐Sarcopenia demonstrating a borderline significance (HR = 1.50, 95% CI: 0.99–2.25, *p* = 0.054). In contrast, the effect of sarcopenia in male patients was weaker and did not reach statistical significance.

#### 3.5.2 Age‐Dependent Effects

A significant interaction was observed between sarcopenia and age, with patients younger than 80 years experiencing a substantially higher mortality risk. In this subgroup, PMA‐Sarcopenia was associated with an HR of 1.88 (95% CI: 1.18–2.99, *p* = 0.008) and PMI‐Sarcopenia with an HR of 1.92 (95% CI: 1.21–3.05, *p* = 0.006). Conversely, in patients aged ≥ 80 years, the association was weaker and nonsignificant (HR = 1.22, *p* = 0.293 for PMA; HR = 1.24, *p* = 0.262 for PMI).

#### 3.5.3 Impact of Cardiac Function

Again, a strong effect of sarcopenia was seen in patients with reduced LVEF (< 40%). PMA‐Sarcopenia showed an HR of 2.75 (95% CI: 1.40–5.38, *p* = 0.003), while PMI‐Sarcopenia had an HR of 1.93 (95% CI: 0.95–3.92, *p* = 0.070), not reaching statistical significance. Further, no statistical differences could be observed regarding SVi modifications neither in PMA nor in PMI.

#### 3.5.4 Metabolic and Renal Function

Chronic kidney disease (CKD) emerged as a major modifying factor. Patients with creatinine ≥ 1.5 mg/dL showed the highest mortality risk, with PMA‐Sarcopenia HR = 3.05 (95% CI: 1.35–6.87, *p* = 0.007) and PMI‐Sarcopenia HR = 3.25 (95% CI: 1.52–6.92, *p* = 0.002). Regarding BMI, sarcopenic patients with BMI ≥ 25 kg/m^2^ had a significantly increased mortality risk (HR = 1.68, *p* = 0.017 for PMA; HR = 1.66, *p* = 0.011 for PMI), while the association was nonsignificant in BMI < 25 kg/m^2^.

#### 3.5.5 Haematological Parameters

Anaemia was a strong modifier of the sarcopenia‐mortality association, with significant effects seen in anaemic patients. PMA‐Sarcopenia had an HR of 1.84 (95% CI: 1.22–2.78, *p* = 0.003), while PMI‐Sarcopenia showed an HR of 1.71 (95% CI: 1.11–2.64, *p* = 0.014). In contrast, no significant association was found in nonanaemic patients.

## 4 Discussion

Sarcopenia has increasingly been recognized as a marker associated with health outcomes in older adults, yet its role in long‐term survival after TAVI remains critically underexplored. With this study, we not only confirm the deleterious impact of sarcopenia on post‐TAVI prognosis but also redefine its clinical significance by demonstrating its disproportionate effect in younger patients, its complex interplay with renal dysfunction and anaemia and its persistence as an independent risk factor even after cardiovascular risk adjustment. These findings challenge the traditional view of sarcopenia as a passive marker of frailty and support its consideration as an active contributor to post‐TAVI mortality, emphasizing the need for its urgent integration into clinical decision‐making and rehabilitation strategies.

### 4.1 Sarcopenia as a Determinant of Long‐Term Post‐TAVI Mortality: A Paradigm Shift

The notion that muscle mass depletion predicts poor outcomes is not novel, yet most prior studies have focused on its short‐term impact [16]. Several reports have linked reduced skeletal muscle area with prolonged hospital stays and early procedural complications [17], but data on long‐term survival remain scarce. Our results build on these observations, demonstrating that sarcopenia remains a potent predictor of mortality even 5 years after TAVI, with HR exceeding 1.5 in adjusted models. This underscores the idea that muscle mass is not merely a procedural risk marker but may reflect a fundamental determinant of long‐term physiological resilience. Our findings contrast with those of Bate et al. [18], who observed a weaker association between sarcopenia and mortality at 1‐year post‐TAVI but did not extend follow‐up beyond that point. Similarly, the study by Kofler et al. [19], which linked CT‐derived psoas muscle measurements to early outcomes, did not account for the long‐term trajectory of sarcopenia after the procedure. By extending the follow‐up to 5 years, our study provides additional evidence suggesting that muscle mass at baseline is not merely a static marker of frailty but may be a dynamic determinant of survival over time, independent of traditional cardiovascular risk factors.

While previous studies have demonstrated the prognostic value of CT‐derived muscle area in other clinical settings—such as cancer [20] and chronic obstructive pulmonary disease [21]—its integration into cardiovascular risk stratification remains underutilized. Given our findings, we advocate for incorporating sarcopenia assessment into standard pre‐TAVI evaluations.

### 4.2 The Unexpected Age Paradox: Why Sarcopenia Matters More in Younger Patients

One of the most striking observations in our study is that the impact of sarcopenia on mortality is significantly greater in patients younger than 80 years compared to their older counterparts. This challenges previous research, which has largely regarded sarcopenia as an inevitable age‐related phenomenon [1, 22] and suggests that muscle depletion in younger individuals is a marker of accelerated physiological decline rather than chronological aging itself. Our study further highlights that sarcopenia in younger patients is not simply an age‐related condition but a pathological state that confers a disproportionately high risk. This raises a fundamental question: Are we underestimating the impact of sarcopenia in “younger” cardiovascular patients?

Several potential mechanisms could explain this phenomenon. While younger TAVI patients might be expected to have a fewer overall burden of age‐related comorbidities, the fact that they undergo TAVI rather than surgical aortic valve replacement (SAVR) suggests that they are, in fact, highly multimorbid [23]. This selection bias implies that younger patients undergoing TAVI are not necessarily healthier but rather have a distinct and complex profile of risk factors that preclude them from surgery [24]. In this context, sarcopenia may serve as a pivotal marker of systemic physiological vulnerability, further amplifying the risk of poor outcomes after TAVI.

While older patients may have developed compensatory physiological adaptations to mitigate the effects of muscle loss over time [25], younger patients with sarcopenia may lack such adaptive responses, leading to a more rapid functional decline and heightened susceptibility to post‐TAVI physiological stressors. Additionally, younger patients may be more affected by sarcopenia because age‐related muscle loss in this group is not part of the normal aging trajectory but rather reflects a pathologic, accelerated catabolic state. Muscle atrophy in younger individuals is more likely to indicate underlying systemic inflammation, chronic disease or cachexia‐related mechanisms, all of which may exacerbate postoperative vulnerability [22, 26]. In contrast, older patients may have adapted metabolically and functionally to gradual muscle decline over the years, whereas the same degree of depletion in a younger person signals a more abrupt physiological breakdown. The combination of sarcopenia and multimorbidity may create a particularly unfavourable prognosis in this group, as both factors contribute to diminished physiological reserves and impaired recovery capacity.

These findings underscore the need for a critical reassessment of current risk stratification models. Traditional frailty indices, which are often tailored to the oldest patients, may not adequately capture the true burden of muscle depletion in younger individuals.

### 4.3 The Female Paradox: When the Survival Advantage Disappears

It is well‐established that female TAVI patients generally exhibit better survival than their male counterparts. This phenomenon has been attributed to factors such as smaller valve sizes, lower rates of coronary artery disease and a more favourable haemodynamic response post‐TAVI [14, 27]. However, our study reveals that this protective effect is weakened in the presence of sarcopenia.

Consistent with the findings of Mamane et al. [15] and Van Mourik et al. [28], who reported sex‐based differences in muscle mass distribution, our data suggest that female patients with sarcopenia face a disproportionately high risk of mortality compared to their male counterparts. This discrepancy may be attributed to several factors, including lower baseline muscle reserves, hormonal differences in muscle metabolism or distinct inflammatory responses. Interestingly, while male patients may demonstrate greater resilience to the metabolic consequences of sarcopenia, females seem to experience a more pronounced decline once a critical threshold is crossed. These sex‐specific differences are underpinned by fundamental aspects of muscle physiology. Women generally have lower baseline muscle mass, reduced testosterone levels and a different distribution of Type I and Type II muscle fibres, which may impact both muscle strength and regenerative capacity [29]. Hormonal changes after menopause—particularly the decline in oestrogen—are associated with increased muscle catabolism and diminished anabolic signalling. These physiological changes may make older female patients especially susceptible to the adverse outcomes of sarcopenia, thereby weakening the survival advantage typically observed in female TAVI patients. Beyond a certain threshold of muscle loss, survival rates decline sharply, highlighting the critical impact of sarcopenia in this population [30]. These findings underscore the importance of establishing sex‐specific sarcopenia thresholds and highlight the potential benefits of early interventions, such as nutritional support and resistance training, particularly for female TAVI candidates.

### 4.4 Beyond the Heart: The Interplay Between Sarcopenia, Renal Dysfunction and Anaemia

Renal function in this cohort was assessed using serum creatinine, the standard clinical marker for kidney function. However, this choice warrants careful interpretation in the context of sarcopenia. Because creatinine is a byproduct of muscle metabolism, its levels are inherently dependent on muscle mass, rather than kidney function alone. As a result, sarcopenic patients, who have reduced muscle mass, also exhibit lower baseline creatinine levels, which may lead to an underestimation of CKD prevalence [31]. Consequently, the true burden of renal dysfunction in our population is likely higher than reported, and conventional creatinine‐based assessments may fail to capture the full extent of kidney impairment in sarcopenic patients [32].

Beyond its methodological implications, this interplay between sarcopenia and CKD has profound clinical consequences. In our study, patients with both sarcopenia and CKD exhibited a threefold increase in mortality risk, reinforcing the growing recognition that muscle wasting and renal dysfunction are not independent phenomena but interconnected pathophysiological processes [33]. These results align with those of Jiang et al. [34], who demonstrated that in CKD populations, sarcopenia accelerates systemic catabolism, worsens metabolic dysregulation and heightens cardiovascular risk. Our study extends this understanding by showing that this harmful synergy persists in post‐TAVI patients, highlighting the need for integrated management strategies that address both muscle loss and renal function deterioration simultaneously, rather than treating them as separate entities.

Similarly, the combined effect of anaemia and sarcopenia on mortality in our cohort suggests that low muscle mass may impair erythropoiesis and oxygen delivery, further amplifying systemic frailty [35, 36]. From a physiological perspective, anaemia and sarcopenia are mechanistically linked: muscle tissue is highly oxygen‐dependent, and chronic low haemoglobin levels can impair mitochondrial function [37], reduce endurance capacity and trigger further muscle wasting. At the same time, skeletal muscle itself is a source of erythropoietin signalling and iron metabolism regulation. Thus, muscle atrophy may not only be a consequence but also a contributor to impaired erythropoiesis, creating a self‐perpetuating cycle of frailty. Given that anaemia is a modifiable risk factor, future trials should explore whether early correction of anaemia could mitigate the adverse impact of sarcopenia on post‐TAVI survival.

### 4.5 Clinical Implications: Time to Move Muscle to the Center of Cardiovascular Care

There are several potential clinical implications of these findings:

- Routine CT‐based sarcopenia assessment should be incorporated into standard TAVI evaluations as a prognostic marker.
- There is ample evidence to suggest that sarcopenia is not just a marker of frailty and poor postprocedural outcomes but an independent and causative risk factor.
- Since sarcopenia is modifiable, it could be seen as a potential target for early therapeutic intervention in patients before and/or after TAVI—or even earlier in the course of the disease.
- We propose that a shift in paradigm in the integrative management of patients with severe aortic valve stenosis may be required, moving from a purely haemodynamic approach to one that integrates also multiple skeletal muscle‐targeted interventions into cardiovascular care. Given that muscle mass is trainable, structured resistance training [38, 39], protein supplementation [39] and novel pharmacological approaches (e.g., myostatin inhibitors) [40] could represent a new frontier in cardiovascular (p)rehabilitation.
- If sarcopenia is indeed an independent determinant rather than a mere marker of poor outcome, failing to address it may mean losing a critical window for outcome optimization. However, the specific nature of such measures—and particularly the right timing—must be clarified by future studies.

### 4.6 Limitation

Despite its strengths, this study has certain limitations that should be acknowledged. First, the retrospective single‐centre design may limit the generalizability of the findings to other TAVI populations. However, the large sample size and systematic long‐term follow‐up enhance the interpretability of the results. Second, sarcopenia was assessed using CT‐derived psoas muscle measurements, which, while widely validated, do not account for muscle function or fat infiltration. Future studies incorporating dual‐energy X‐ray absorptiometry (DEXA), bioelectrical impedance analysis (BIA) or handgrip strength could provide a more comprehensive evaluation of sarcopenia. Third, we were unable to assess dynamic changes in muscle mass post‐TAVI, which would be crucial for understanding whether sarcopenia progression or reversal impacts long‐term outcomes. We acknowledge the importance of longitudinal follow‐up data on sarcopenia improvement or deterioration. Future prospective and ideally multicentre studies are warranted to validate our findings and explore whether interventions targeting sarcopenia can improve patient selection and outcomes after TAVI. As with all observational studies, residual confounding cannot be entirely excluded, although rigorous multivariate adjustments and sensitivity analyses were applied to strengthen the reliability of our findings. Finally, the results of our study relate to patients with severe aortic valve stenosis and are not applicable to patients with other types of structural heart diseases.

## 5 Conclusion

**FIGURE 6:**
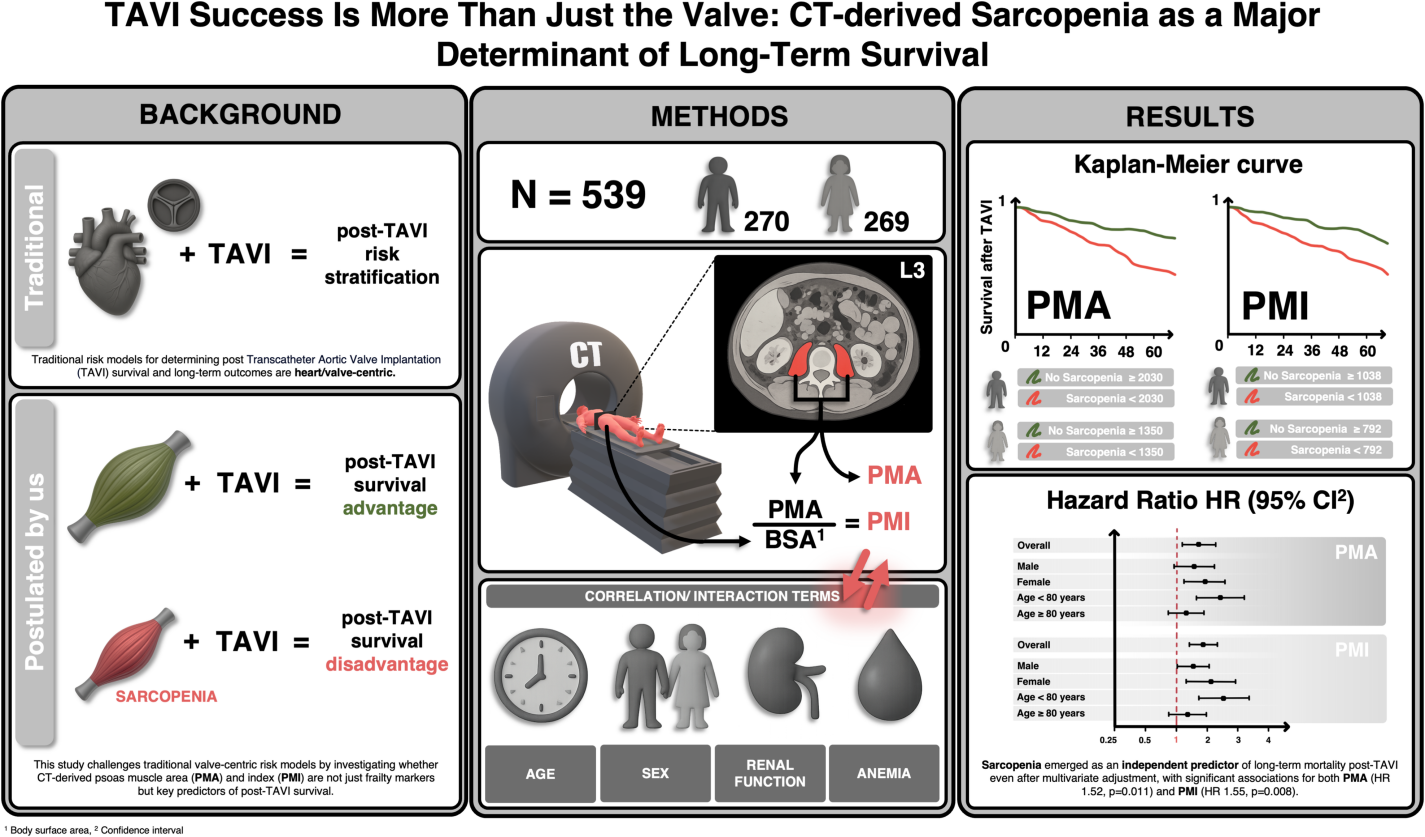
Graphical abstract. Abbreviations: BSA, body surface area; CI, confidence interval; HR, hazard ratio; PMA, psoas muscle area; PMI, psoas muscle area index; TAVI, transcatheter aortic valve implantation.

This study establishes CT‐driven sarcopenia as a powerful, independent predictor of long‐term mortality in TAVI patients, with particularly pronounced effects in younger patients, females and those with renal dysfunction and anaemia. These findings challenge current cardiovascular risk models and underline the need to consider integrating nutrition‐, exercise‐ and/or medication‐based muscle‐targeted interventions into the routine care of patients with severe aortic valve stenosis. Sarcopenia is not merely a marker of frailty or a sedentary lifestyle, but very probably an actionable target for improving survival—possibly also in patients with other structural heart diseases. Recognizing this is not just an academic exercise; it is a call to revolutionize how we assess and treat cardiovascular patients in the era of structural heart interventions. A visual summary of these findings and their clinical implications is provided in Figure 6.

## Ethics Statement

The study was approved by the Ethics Committee of the state of Salzburg, Austria (EK‐Nr. 1082/2024). All data collection and handling complied with the ethical guidelines set forth in the Declaration of Helsinki and the ICH‐GCP (International Council for Harmonization of Technical Requirements for Pharmaceuticals for Human Use—Good Clinical Practice) standards. Due to the retrospective nature of the study, the Ethics Committee of Salzburg waived the need for written informed consent.

## Conflicts of Interest

Nikolaos Schörghofer, Christoph Knapitsch, Gretha Hecke, Nikolaus Clodi, Lucas Brandstetter, Crispiana Cozawicz, Matthias Hammerer, Klaus Hergan, Uta C. Hoppe, Bernhard Scharinger and Elke Boxhammer declare that the research was conducted in the absence of any commercial or financial relationships that could be construed as a potential conflicts of interest.
